# Maxdenominator Reweighted Sparse Representation for Tumor Classification

**DOI:** 10.1038/srep46030

**Published:** 2017-04-10

**Authors:** Weibiao Li, Bo Liao, Wen Zhu, Min Chen, Li Peng, Xiaohui Wei, Changlong Gu, Keqin Li

**Affiliations:** 1 College of Information Science and Engineering, Hunan University, Changsha, Hunan 410082, China; 2 Department of Computer Science, State University of New York, New Paltz, New York 12561, USA

## Abstract

The classification of tumors is crucial for the proper treatment of cancer. Sparse representation-based classifier (SRC) exhibits good classification performance and has been successfully used to classify tumors using gene expression profile data. In this study, we propose a three-step maxdenominator reweighted sparse representation classification (MRSRC) method to classify tumors. First, we extract a set of metagenes from the training samples. These metagenes can capture the structures inherent to the data and are more effective for classification than the original gene expression data. Second, we use a reweighted 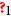 regularization method to obtain the sparse representation coefficients. Reweighted 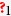 regularization can enhance sparsity and obtain better sparse representation coefficients. Third, we classify the data by utilizing a maxdenominator residual error function. Maxdenominator strategy can reduce the residual error and improve the accuracy of the final classification. Extensive experiments using publicly available gene expression profile data sets show that the performance of MRSRC is comparable with or better than many existing representative methods.

Accurate tumor classification is beneficial for cancer treatment. A tumor can be classified as benign, premalignant, or malignant, of which only a malignant tumor can be called cancer. Thus, a reliable and precise classification of tumors is valuable for accurate diagnosis.

Traditional histopathological approach classifies tumors by microscopic tissue examination. However, this process fails to classify many cancer cases. Many classification methods using gene expression profile data have been adapted to classify tumors. Golub *et al*.^1^ successfully distinguished acute myeloid leukemia (AML) from acute lymphocytic leukemia (ALL) using gene expression profile data. Furey *et al*.^2^ classified cancer tissue samples by support vector machines (SVMs) with gene expression profile data. Bhattacharjee *et al*.^3^ precisely classified human lung humors. Ship *et al*.^4^ predicted lymphoma by gene expression profile data and supervised machine learning. Huang *et al*.^5^ used independent component analysis (ICA)-based penalized discrimination method to classify tumors. Ghosh *et al*.^6^ used least absolute shrinkage and selection operator method to classify and select biomarkers in genomic data. All these methods will suffer from overfitting when used to classify tumors.

Sparse representation has been attracting much attention because of the progress in 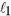 norm minimization-based methods, such as basis pursuit^7^ and compressive sensing theory^8^,^9^,^10^. Wright *et al*.^11^ proposed a sparse representation-based classification (SRC) method for face recognition. Common two-stage machine learning methods initially create a training model for testing, and then the samples are tested. By contrast, SRC uses a sparse linear combination of the training samples to represent the testing sample. In this model, a 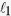 norm least square method^12^ is applied to search for the sparse representation coefficient which will decide the type of the test sample. This essential characteristic would prevent the SRC to perform the training and testing steps, reducing the overfitting problem. Hang *et al*.^13^ used SRC to classify tumors and obtained very good experimental results.

However, the original training samples of gene expression profile data may be not efficient to represent the input testing samples. Brunet *et al*.^14^ demonstrated that metagenes can recover meaningful biologic information from gene expression profile data. A metagene which can cover the structure inherent to the data is a linear combination of the gene expression profiles of samples. Metagenes can be obtained using singular value decomposition (SVD), principal component analysis (PCA), and nonnegative matrix factorization (NMF). Brunet *et al*.^14^ discovered the metagenes by NMF and used these metagenes to cluster the samples, yielding very good results. Zhen *et al*.^15^ used SVD or NMF to capture the metagenes from the gene expression profile data. In the current study, we determined the metagenes using SVD. Several studies have reported on the extraction of the metagenes. NMF was used in ref. 14 to decompose gene expression patterns as an additive combination of a few metagene patterns. The NMF metagenes could overlap and thus expose the participation of a single gene in multiple pathways or processes. ICA was applied in ref. 16 to gene expression data, and a linear model, which was termed “expression modes,” was derived. In this model, the expression of each gene is a linear function of the expression modes. The dominant expression modes were experimentally found to be related to distinct biological function, such as the phase of the cell cycle or the mating response. SVD was applied in ref. 17 to transform genome-wide expression data from “genes” × “arrays” space to reduced diagonalized “eigengens” × “eigenarrays” space, where the eigengenes (or metagenes) are unique orthonormal superposition of the genes (or samples). SVD was applied to capture the weighted metagenes^18^, then the test sample is represented as the linear combination of these weighted metagenes. These analytical results show that using metagenes to replace gene expression data can produce better results for classification.

In addition, 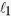 norm is just as a replacement of the 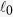 norm in the objective function of sparse representation. However, 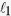 and 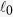 norms significantly differ. According to the definitions of 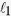 and 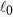 norms, larger coefficients are penalized more heavily in the 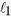 norm than smaller coefficients, contrary to the more democratic penalization of the 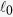 norm. Candès *et al*.^19^ addressed this problem by proposing a reweighted 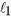 norm minimization method for signal recovery. In the current study, we also use the reweighted 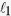 norm minimization strategy to find the sparse representation coefficients.

We propose a maxdenominator residual error function which takes full advantage of the linear relation between test sample and metagenes to capture the classification from the sparse representation coefficients.

We first extract a set of metagenes from the training samples. Each testing sample is represented as a linear combination of metagenes, and the linear combination coefficients of metagenes are captured by reweighted 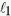 norm minimization strategy. Then, we use a maxdenominator residual error function to obtain the classification result. Our method is named as Maxdenominator Reweighted Sparse Representation Classification (MRSRC).

The rest of this paper is organized as follows. In the Result Section, the experiments will be presented, and the performance of the proposed method will be compared with those of several common methods for tumor classification. In the Discuss Section, the results are discussed, and conclusions are drawn. Finally, we will describe the metagene model and briefly review SRC. We will also introduce the reweighted sparse representation method and the maxdenominator residual error function. We will also describe the details of MRSRC, including its object function and the solution algorithm in the Method Section.

## Results

In this section, we run an experiment to evaluate the performance of the proposed MRSRC method. The experiment is divided into five parts. The first part involves the two-class classification given in the Two-Class Classification Section. The second part is the multi-class classification given in the Multiclass Classification Section. The third part comprises the different feature dimensions given in the Experiment with different number of genes Section. The fourth part involves the cross-validation given in the Comparison of CV performance Section. The fifth part visually presents the sparse representation coefficients given in the Visualization of Sparse Representation Coefficients Section. The proposed method is compared with several state-of-the art methods, such as the widely used LDA + SVM^20^, ICA + SVM^21^, SRC^13^, and SVD + MRSC^15^, MACE^22^, OTSDF^22^. The former two methods are models based on SVM as a classification and accompanied by feature extraction. We use these two methods as the baseline. The latter two methods are template based and get very good performance for classification of tumors.

**Figure 1:**
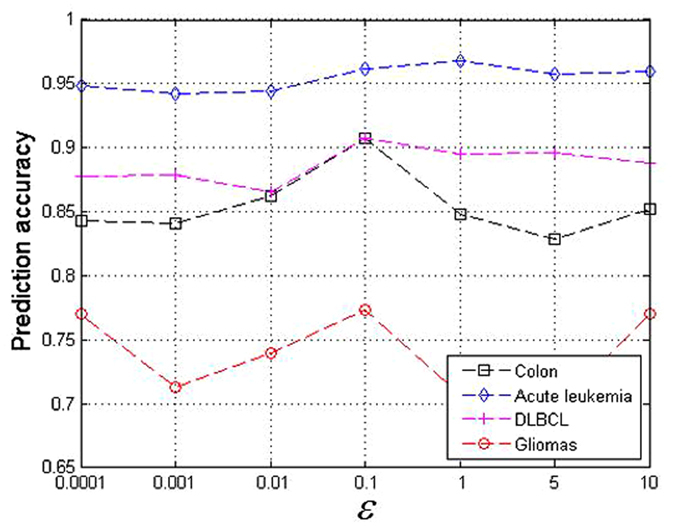
Optimal classification accuracy of MRSRC on four binary class dataset.

In our proposed MRSRC method, three parameters should be set, namely, positive regularization parameter λ, stability parameter *ε*, and maximum reweighted iterations parameter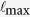. According to a previous study^23^, the value of λ is given as follows:


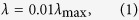



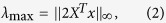


where 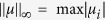 denotes the 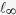 norm of the vector *μ*. For the stability parameter *ε*, as a rule of thumb, *ε* should be slightly smaller than the expected nonzero magnitudes of *θ*. From Fig. 1, we can set *ε *= 0.1 in this paper. Finally, we set the maximum reweighted iterations parameter 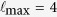. These values were selected because much of the benefit comes from the first few reweighting iterations, and the added computational cost for improved sparse representation coefficients is quite moderate.

The SVM kernel parameters for the LDA + SVM and ICA + SVM algorithms are determined by 10-fold cross validation. In addition, we simply extract *c* − 1 (where *c* denotes the number of classes) new features to train the classifier, because LDA can find a maximum of *c* − 1 meaningful projection vectors in the subspace. Moreover, the determination of the number of independent components of ICA is also an empirically dependent work. Here, we use the same method as suggested by ref. 24 Zheng *et al*. The SRC and MSRC methods also need parameter *λ* to control sparsity. The parameters of methods of MACE and OTSDF are set as recommended by^22^ Wang *et al*.

To avoid the effects of imbalanced training set, we use Balance Division Method (BDM) to divide each original data set into balanced training set and test set. For this BDM, *Q* samples from each subclass are randomly selected to be used for training set, and the rest are used for test set. Here, *Q* must be an integer number. For example, if we set *Q* to 5, then 5 samples per subclass randomly selected are used as training set and the rest are assigned to the test set. Then we evaluate the performance of seven methods on a balanced split data set. We randomly select *p* = 5 to 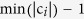 samples per subclass as training set and use the rest for testing to guarantee that at least one sample in each category can be used for test. In the test, *p* denotes the number of training samples per classes, and 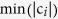 denotes the minimum number of subclass set of samples in the training data. The training/testing are performed 10 times, and the average classification accuracies are presented.

To evaluate the classification performance in imbalanced split training/testing sets, we perform a 10-fold stratified CV on a tumor subtype data sets. All samples are randomly divided into 10 subsets on the basis of stratified sampling: nine subsets are used for training, and the remaining samples are used for testing.

We determine the accuracy, sensitivity, and specificity to measure the performance for a fair experimental evaluation. These metrics are defined as follows:


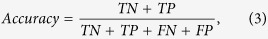



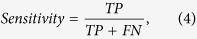



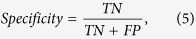


where true positives (TP) and true negatives (TN) denote the ratio of samples that are correctly classified into the positive and negative groups, respectively. False negatives (FN), and false positives (FP) denote the incorrectly classified true positive samples into the negative group and true negative samples into the positive group, respectively.

### Two-Class Classification

In this section, four publicly available microarray data sets are used to compare the performance of the methods. These data sets are acute leukemia data set^1^, colon cancer data set^25^, gliomas data set^26^, and diffuse large B-cell lymphoma (DLBCL) data set^27^ (please see supplementary information for data sets).

The acute leukemia data set contains 72 samples of 7,129 genes. The target class has 2 states, including 47 acute lymphoblastic leukemia patients and 25 acute myelogenous leukemia patients. The colon cancer data set includes 40 tumor and 22 normal colon tissue samples and contains the expression of 2,000 genes with the highest minimal intensity across 62 tissues. The gliomas data set consists of 50 samples with two subclasses (glioblastomas and anaplastic oligodendrogliomas), and each sample contains 12,625 genes. The DLBCL data set contains 77 samples of 7,129 genes. The target class has 2 states, including 58 diffuse large b-cell lymphoma samples and 19 follicular lymphoma samples. Table 1 provides the details of the data sets.

**Figure 2:**
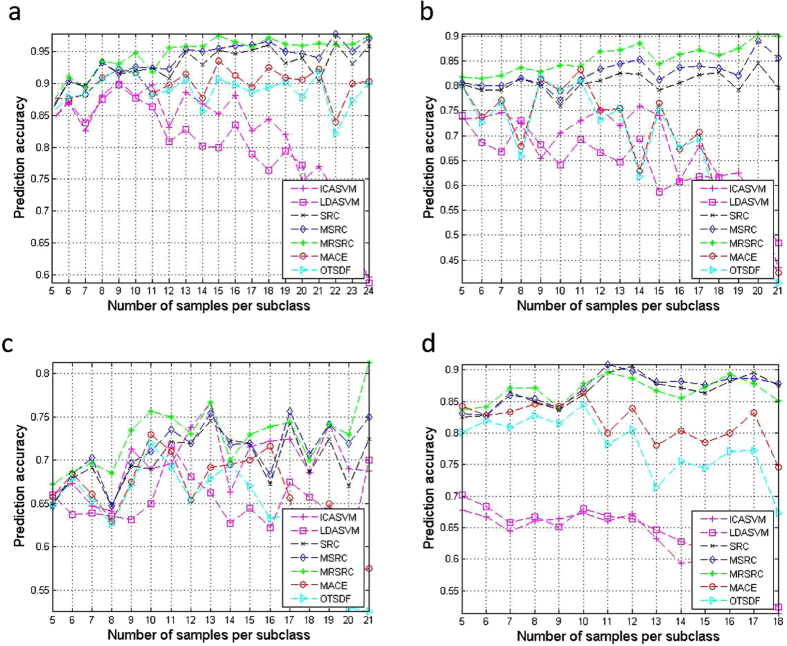
Comparison of prediction accuracy on four binary classification datasets by varying the number of samples from per subclass.

The average prediction accuracies of the classification of the balanced training set and test set are shown in Fig. 2. The MRSRC exhibits encouraging performance. The MRSRC achieves the best classification accuracy in all cases for the colon cancer data (Fig. 2b). Although gliomas are difficult to classify, the proposed approach can still achieve the highest classification accuracy via 21 (81%) samples per subclass used for training (Fig. 2c). MRSRC also achieves relatively high prediction accuracies in most cases in the acute leukemia and DLBCL data sets (Fig. 2a and d). Notably, the classification accuracies of LDA + SVM and ICA + SVM drop quickly as the number of samples considered for training increases. These results are consistent with the observations in the literature^22^.

We illustrate the experimental results by showing the sensitivity and specificity of the proposed MRSCR, SCR, and MSCR in Tables 2 and 3 when the numbers of metagenes per subclass are fixed to 10.

The experimental results and analysis show that the proposed MRSRC is highly competitive for two-class tumor classification.

### Multiclass Classification

In this section, four multiclass data sets are used to further investigate the performance of the MRSRC. The experimental setup is the same as that for the binary classification case. The data sets include the small, round blue cell tumors (SRBCT)^28^, ALL^29^, MLLLeukemia^30^, and LukemiaGloub^1^ (please see supplementary information for data sets).

The SRBCT data set is composed of four types of tumors. This data set includes 83 samples with 2,308 genes. The ALL data set consists of 248 samples with six subclasses. Each sample contains 12,625 genes. The MLLLeukemia data set consists of 72 samples with three subclasses. Each sample contains 12,582 genes. The LukemiaGloub data set consists of 72 samples with three subclasses. Each sample contains 7,129 genes. Table 4 provides details of the data sets.

**Figure 3:**
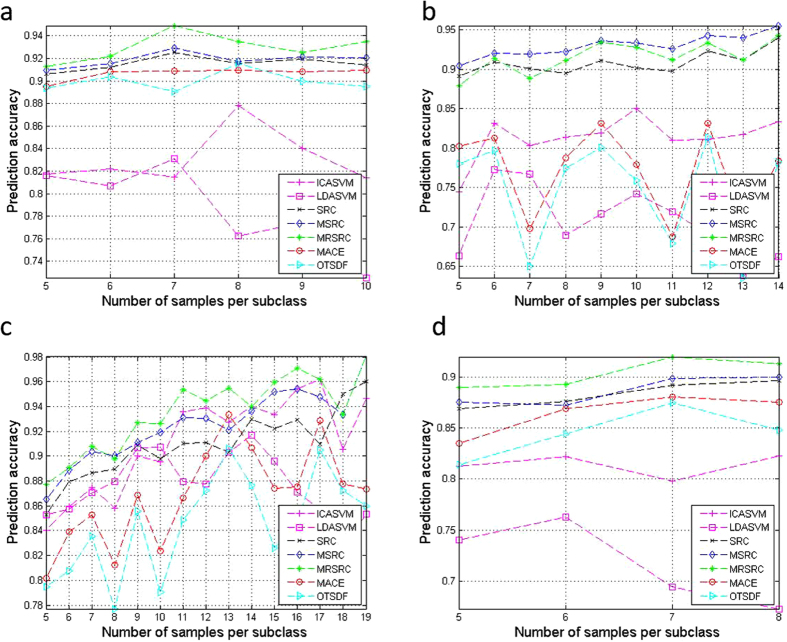
Comparison of prediction accuracy on four multiclass classification datasets by varying the number of samples from per subclass.

The average prediction accuracy of the classification is shown in Fig. 3. The MRSRC achieves the best classification accuracy in all cases in the SRBCT data and LukemiaGloub data (Fig. 3a and d). In the ALL experiments (Fig. 3b), the proposed MRSRC shows no evident advantages over SRC and MSRC. The MRSRC achieves extremely high prediction accuracy in most cases for the MLLLeukemia data set (Fig. 3c). As shown by the data dimensions in the four experiments, the MRSCR offers more advantages and more stability than other methods.

### Experiment with different numbers of genes

In this subsection, we evaluate the performance of the seven methods with different feature dimensions on eight tumor data sets. For the training data, 10 samples per subclass are randomly selected, whereas the remaining samples are used for the test. We perform the test with various numbers of genes, starting from 102 to 352 genes in increments of 5. The top genes are selected from each data set by applying the Relief-F algorithm^31^ to the training set.

**Figure 4:**
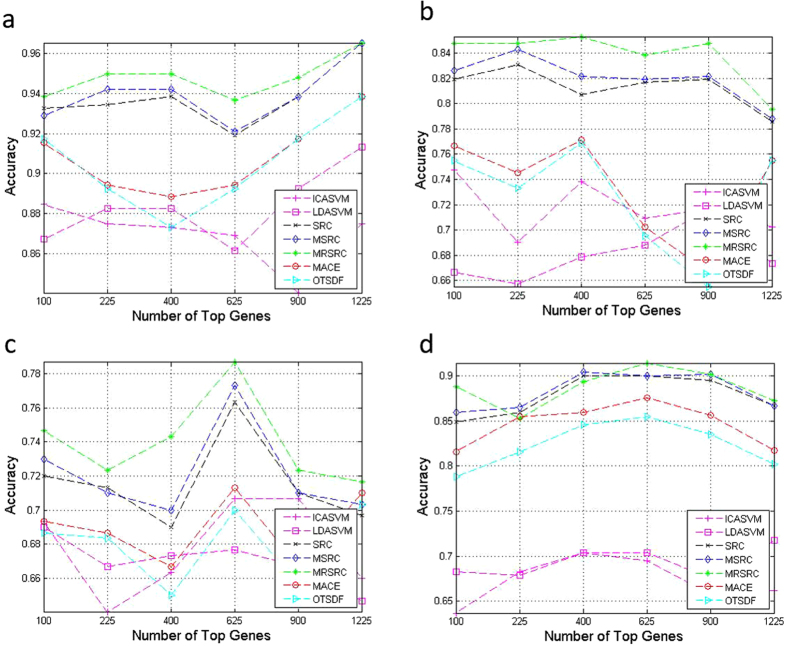
Comparison of accuracy on four binary classification datasets by varying the number of top selected genes.

Figure 4 presents the result of the binary classification. The MRSRC outperforms the other methods in terms of prediction accuracy for all data sets, except for the DLBCL data set (Fig. 4d). The gene selection of the MRSRC is better than that of other methods for the acute leukemia, colon, and gliomas data sets (Fig. 4a,b and c). The MRSRC, SRC, and MSRC share the same curve trend. Evidently, the MRSRC, SRC, and MSRC consistently outperform LDASVM, ICASVM, MACE, and OTSDF in all data sets.

**Figure 5:**
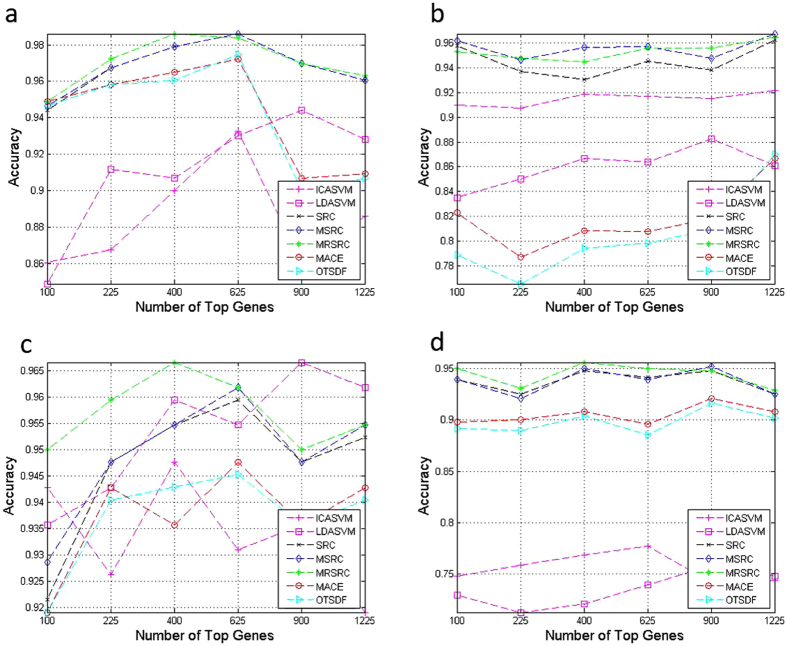
Comparison of accuracy on four multiclass classification datasets by varying the number of top selected genes.

Figure 5 shows the average prediction accuracy of multiclass classification. As shown in Fig. 5, the MRSRC is robust with respect to the number of top-ranked genes. Selection can obviously improve the classification accuracies of all methods in the four data sets. The results show remarkable progress, especially for LDASVM. This result is due to the fact that LDA can capture a large amount of discriminating information in the multiclass classification task and significantly reduce the over-fitting phenomenon in comparison with its performance in the binary classification task.

### Comparison of CV performance

To evaluate the classification performance of the MRSRC, SRC, and MSRC in the imbalanced split training/testing sets, we perform a 10-fold stratified CV on the tumor subtype data set. All samples are randomly divided into 10 subsets on the basis of stratified sampling: nine subsets are used for training, and the remaining samples are used for testing. This evaluation process is repeated 10 times, and the average result is presented. The 10-fold CV results are summarized in Tables 5, 6 and 7.

Table 5 Shows that the MRSRC achieves the best accuracy in the seven data sets. Especially in the case of multiclass classification, the MRSRC shows superior performance in all data sets. Table 6 shows that the MRSRC achieves the highest value in all eight data sets. Table 7 shows that the MRSRC achieves the highest specificity in all the multiclass.

We can conclude that the MRSRC is outstanding in the imbalance split training/testing sets.

### Visualization of Sparse Representation Coefficients

**Figure 6:**
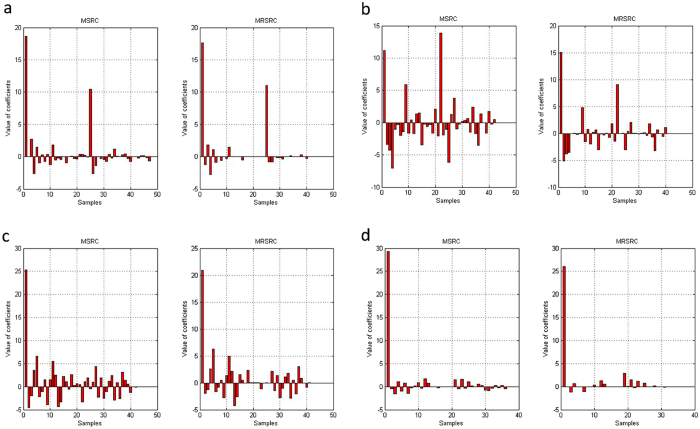
The value of the sparse representation coefficients of MSRC and MRSRC on four binary classification datasets when choosing one sample as test set.

**Figure 7:**
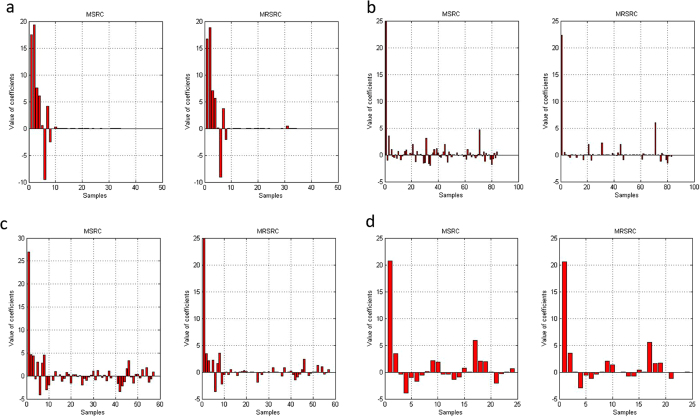
The value of the sparse representation coefficients of MSRC and MRSRC on four multiclass classification datasets when choosing one sample as test set.

To further analyze the results, we compared the value of coefficients of MSRC and MRSRC for the eight data sets. Figures 6 and 7 show the value of the sparse representation coefficients of the training samples. A test samples is represented as the linear relation of metagenes of such training samples. From Figs 6 and 7, we can observe that MRSRC obtains better sparse representation coefficients. MRSRC demonstrates more coefficients, which are equal to zero and are close to the theoretical results.

Figures 6 and 7 Also show that the test sample can be represented as the linear combination of metagenes. The figures illustrate that metagenes can recover meaningful biological information from gene expression profile data and can cover the structure inherent to the data. Each metagene in the data set obtained maximum coefficient, which indicates that the test sample is very similar to the metagene.

## Discussions

Sparse representation-based methods (SRC, MSRC, and MRSRC) consistently outperform the model-based methods (LDASVM and ICASVM) and the template-based methods (MACE and OTSDF) in most experiments. This result is probably due to the small sample size problem for model-based strategies. Sparse representation-based methods perform well even when five samples per subclass were considered for training and the rest for testing. Moreover, MRSRC outperforms SRC and MSRC in most cases, which implies that reweighted and maxdenominator strategies can contribute to improve sparse solution and obtain better classification results. Additionally, gene selection can enhance the accuracy of classification using all datasets. Gene expression profiling involves data with high dimensionality, and exclusion of the redundant is critical for classification.

In this paper, we propose a new sparse representation-based method for classifying tumors. This method adapts reweighted strategy to balance 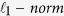. The reweighted strategy contributes to capture sparse solution, which is used for classification. Classification is achieved by a maxdenominator residual error function, which takes full advantage of the linear relations between the testing sample and metagenes extracted using SVD from the training samples. We also compare the performance of MRSRC with those of two sparse representation-based methods, two model-based methods and two template-based methods using eight tumor expression datasets. The results have shown the superiority of MRSRC and validated the effectiveness and efficiency of MRSRC in tumor classification.

MRSRC exhibits stable performance with respect to different training sample sizes compared with the other four methods. The properties of this reweighted sparse representation algorithm should be investigated further. Thus, we will extend the algorithm with dimensionality reduction in our future studies.

## Methods

### Metagenes of Gene Expression File Data

Metagenes of gene expression data are captured by mathematical operation on the original sample data. These mathematical operations include SVD, PCA, NMF, ICA, or other linear or nonlinear models. Essentially, mathematical operation on gene expression data contributes to highlight particular biological functions, recover meaningful biological information, capture alternative structures inherent to the data, provide biological insight, reduce noise, and compress the data in a biologically sensible way^14^,^15^,^16^,^17^,^18^. Another viewpoint presented in ref. 15 shows that the gene expression pattern can be approximately represented as linear combination of these metagenes.

Assuming that *X* ∈ *R*^*m×n*^ is a training sample set of gene expression file data, then matrix *X* has the following approximation by mathematical operation:


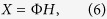


where Φ is a metagene matrix with size of *m* × *p*, and each of the *p* columns are regarded as a metagene. Matrix *H* is a pattern matrix with size of *p* × *n*, and each of the *n* columns are treated as the metagene expression pattern of the corresponding sample. We will use Φ, instead of *X*, for the classification.

Several studies have reported on the extraction of the metagenes. NMF was used in ref. 14 to decompose gene expression patterns as an additive combination of a few metagene patterns. The NMF metagenes could overlap and thus expose the participation of a single gene in multiple pathways or processes. ICA was applied in ref. 16 to gene expression data, and a linear model, which was termed “expression modes,” was derived. In this model, the expression of each gene is a linear function of the expression modes. The dominant expression modes were experimentally found to be related to distinct biological function, such as the phase of the cell cycle or the mating response. SVD was applied in ref. 17 to transform genome-wide expression data from “genes” × “arrays” space to reduced diagonalized “eigengens” × “eigenarrays” space, where the eigengenes (or metagenes) are unique orthonormal superposition of the genes (or samples). SVD was applied to capture the weighted metagenes^18^, then the test sample is represented as the linear combination of these weighted metagenes.

These analytical results show that using metagenes to replace gene expression data can produce better results for classification.

### Sparse Representation for the Classification of Testing Tumor Samples

SRC has been successfully applied to tumor classification in refs 13 and 15. The experimental results showed that SRC is quite competitive in classifying tumors. We simply introduce several notations of this model to enhance the understanding of the SRC. We assume that 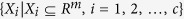 is a set of training samples, and 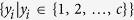 is labeled corresponding to *X*_*i*_, where *m* is the dimensionality of samples, and *c* is the number of classes. The *jth* class training samples *X*_*j*_ can be presented as columns of a matrix 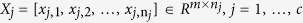, where *x*_*j,I*_ is a sample of the *jth* class, and *n*_*j*_ is the number of the *jth* class training samples. Thus, a training sample matrix *X* can be obtained, as follows:


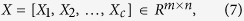


where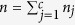.

Then, we use SVD to obtain the metagenes of each class. We factorize each class training sample matrix *X*_*i*_, as follows:


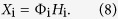


We can derive the metagenes from the *c* classes after computing the metagenes Φ_*i*_ of each class, as follows:


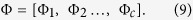


Given a test sample *x* ∈ *R*^*m*^, according to SRC, x can be represented as the linear combination of metagenes of all training samples, as follows:


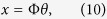


where *θ* ∈ *R*^*n*^ is the sparse representation coefficients. The *x* can also be represented in the form of an equation, as follows:


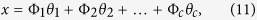


where 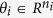 is the coefficient vector corresponding to the *ith* class training samples. Ideally, if *x* belongs to the *jth* class, then *θ* has the following form:


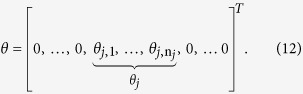


Equation (12) indicates that only the entries corresponding to the *jth* training sample *X*_*j*_ are not zeros. Then, *x* can be finally expressed as follows:


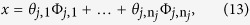


where Φ_*j,i*_ is a metagene of the *jth* class training samples, and *n*_*j*_ is the number of the *jth* class training samples.

Then, the key problem becomes the calculation of the sparse representation vector *θ*. Only a fraction of entries are not zeros, so *θ* is expected to be sparse. Several excellent methods have been put forward in the theory of sparse representation and compressive sensing^8^,^9^,^10^.

### Reweighted Sparse Representation

The sparse representation coefficients of a sample *x* ∈ *R*^*m*^ can be obtained mathematically by solving the combinatorial optimization problem, as follows:


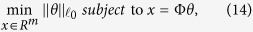


where 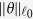 is the 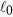 norm. However, the optimization problem given by Equation (14) is NP-hard and generally impossible to solve efficiently, because its solution usually requires an intractable combinatorial search^32^.

A common alternative to the combinatorial optimization problem is to solve the following convex problem:


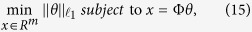


where 
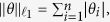
 is the 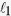 norm. Contrary to Equation (14) and (15) can actually be recast as a linear program and can be solved efficiently^33^. The distinction between Equations (14) and (15) is the choice of objective function, with the latter using 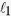 norm as a proxy for the literal 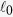 norm sparsity count. However, a key difference exists between the 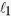 and 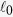 norms. As mentioned earlier, the coefficients are penalized when they are imbalanced in the 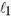 norm, contrary to the more equal penalization in the 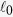 norm.

First, we consider the following weighted 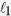 minimization problem to resolve the important issues:


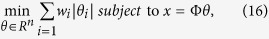


where *w*_*i*_ is the positive weight. Similar to Equation (15) and (16) is convex and can be recast as a linear program. For convenience, Equation (16) can be rewritten as follows:


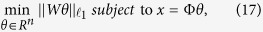


where *W* is a diagonal matrix with *w*_1_ … *w*_*m*_ on the diagonal and zeros elsewhere.

Equation (17) can be viewed as a relaxation of the following weighted 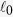 minimization problem:


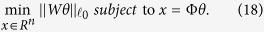


If the solution to Equation (14) is unique, then the mathematical solution to Equation (18) is unique, and the weights do not vanish^19^. However, this situation has changed for the corresponding 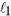 relaxations Equations (15) and (16). These equations will generally have different solutions. Thus, how to set the weights (*w*_*i*_) as free parameters in the convex relaxation is important to improve signal reconstruction.

We set the weights (*w*_*i*_) inversely to the magnitude of *θ*_*i*_, as follows:


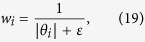


where parameter *ε* > 0 to provide stability and ensure that a zero-valued component in *θ*_*i*_ does not strictly prohibit a nonzero estimate at the next step. Thus, *w*_*i*_*θ*_*i*_ ≈ 1 and 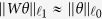. Thus, Equations (14) and (15) present slight difference.

An iterative algorithm that alternates between estimating *θ* and redefining the weights is proposed in ref. 19. We have an updated algorithm of *θ*_*i*_ and *w*_*i*_ as follows:

1. The initial value of iteration count 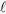 is set to zero, and weights are set to 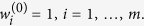


2. The weighted 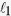 minimization problem is solved as follows:


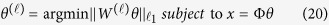


3. The weights are updated, such that for each 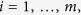



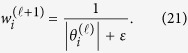


4. When 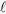 attains a specified maximum number of iterations 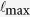 or Equation (20) is terminated upon convergence. Otherwise, 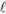 is incremented, and step 2 is reiterated.

In step 4, the parameter of 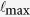 is the maximum number of reweighted iterations.

### Maxdenominator Residual Error Function

The sparse representation coefficient *θ* can be obtained using the iterative algorithm. Ideally, *θ* has a form similar to Equation (12) with nonzero entries corresponding to the class of the testing sample. However, modeling error and noise will inevitably lead to small nonzero entries corresponding to multiple object classes^11^. For a more robust classification, *x* is classified based on how well *x* can be reconstructed using the coefficients from each class^34^. For class *i*, we define a characteristic function *δ*_*i*_, as follows:


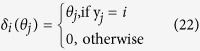


where *y*_*j*_ is the label of training sample *X*_*j*_. This function is used to obtain the ideal coefficients of samples belonging to class *i* and a new vector, as follows:


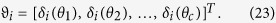


In addition, the test sample is represented as the linear combination of the metagenes in sparse representation. Thus, a metagene from the same class of the test sample is very similar to the test sample. The sparse representation coefficient of this metagene is relatively large. Then, the relatively large coefficient is used to reduce the residual error.

Finally, a maxdenominator residual error function is defined, as follows:


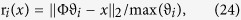


where 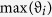 yields the max coefficients of 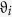. Thus, the label 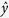 of *x* can be estimated by minimizing the following formula:


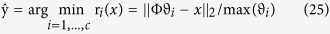


The truncated Newton interior-point method^23^ is used to solve the optimization problem in Equation (17). The solution was performed using the 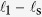 Matlab package available online (https://stanford.edu/~boyd/l1_ls).

The complete classification algorithm of MRSRC is as follows:

**Input**: Training samples 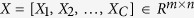


Testing samples *x* ∈ *R*^*m*^

**Output**: Label 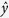 of *x*

Procedure

1. Columns of *X* are normalized to have unit 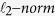.

2. The metagenes of every class are extracted by SVD.

3. The initial value of iteration count 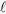 is set to zero, and weights are 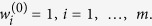


4. The weighted 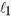 minimization problem Equation (17) is solved to obtain the coefficient vector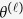.

5. The weights are updated according to Equation (21), that is, for each *i* = 1, …, *m*,.

6. When 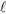 attains a specified maximum number of iterations 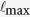 or Equation (20) is terminated upon convergence. Otherwise, 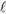 is incremented, and step 2 is reiterated.

7. Count 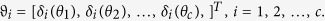


8. The *c* classes residual errors are calculated, that is, 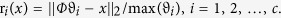


9. The label 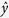 of *x* is estimated according to residual errors.

## Additional Information

**How to cite this article**: Li, W. *et al*. Maxdenominator Reweighted Sparse Representation for Tumor Classification. *Sci. Rep.* **7**, 46030; doi: 10.1038/srep46030 (2017).

**Publisher's note:** Springer Nature remains neutral with regard to jurisdictional claims in published maps and institutional affiliations.

## Supplementary Material

Supplementary Information

Supplementary Dataset 1

Supplementary Dataset 2

Supplementary Dataset 3

Supplementary Dataset 4

## Figures and Tables

**Table 1:** The descriptions of four data sets for two-class classification.

Data set	Classes	Genes	The number of samples
Acute leukemia data	2	7,129	72
Colon cancer data	2	2,000	62
Gliomas data	2	1,2625	50
DLBCL data	2	7,129	77

**Table 2:** The classification sensitivity of two-class classification when the numbers of metagenes per subclass are fixed as 10.

Data set	SRC	MSRC	MRSRC
Acute leukemia data	**94.00%**	93.33%	**94.00%**
Colon cancer data	80.00%	85.83%	**87.50%**
Gliomas data	67.22%	**67.78%**	**67.78%**
DLBCL data	**96.67%**	93.33%	93.33**%**

**Table 3:** The classification specificity of two-class classification when the numbers of metagenes per subclass are fixed as 10.

Data set	SRC	MSRC	MRSRC
Acute leukemia data	93.24%	94.59%	**95.41%**
Colon cancer data	77.33%	84.67%	**85.33%**
Gliomas data	70.00%	**73.33%**	72.50**%**
DLBCL data	88.54%	**89.38%**	88.96%

**Table 4:** The descriptions of four data sets for multiclass classification.

Data set	Classes	Genes	samples
SRBCT data	4	2,308	83
ALL data	6	12,625	248
MLLLeukemia data	3	12,582	72
LukemiaGloub data	3	7,129	72

**Table 5:** 10-fold CV prediction accuracy of eight tumor microarray datasets using different classification methods.

Data set	SRC	MSRC	MRSRC
Acute leukemia data	**83.87%**	**83.87%**	**83.87%**
Colon cancer data	95.83%	97.22%	**98.61%**
Gliomas data	72.00%	72.00%	**78.00%**
DLBCL data	**98.70%**	96.10%	92.21%
SRBCT data	**98.80%**	96.39%	**98.80%**
ALL data	97.98%	97.58%	**98.39%**
MLLLeukemia data	**98.61%**	**98.61%**	**98.61%**
LukemiaGloub data	95.83%	**97.22%**	**97.22%**

**Table 6:** 10-fold CV prediction sensitivity of eight tumor microarray datasets using different classification methods.

Data set	SRC	MSRC	MRSRC
Acute leukemia data	72.73%	**77.27%**	**77.27%**
Colon cancer data	92.00%	92.00%	**96.00%**
Gliomas data	71.43%	71.43%	**78.57%**
DLBCL data	94.74%	94.74%	**100.0%**
SRBCT data	**100.0%**	96.55%	**100.0%**
ALL data	86.67%	86.67%	**93.33%**
MLLLeukemia data	**100.0%**	**100.0%**	**100.0%**
LukemiaGloub data	88.89%	88.89%	**100.0%**

**Table 7:** 10-fold CV prediction specificity of eight tumor microarray datasets using different classification methods.

Data set	SRC	MSRC	MRSRC
Acute leukemia data	**90.00%**	87.50%	87.50%
Colon cancer data	97.87%	**100.0%**	**100.0%**
Gliomas data	72.73%	72.73%	**77.27%**
DLBCL data	**100%**	96.55%	89.66%
SRBCT data	**100%**	**100%**	**100%**
ALL data	98.71%	**99.14%**	**99.14%**
MLLLeukemia data	**100%**	**100%**	**100%**
LukemiaGloub data	**100%**	**100%**	**100%**
