# Supplementary material for: Maxdenominator Reweighted Sparse Representation for Tumor Classification
Source: Sci Rep. 2017 Apr 10;7:46030. doi: 10.1038/srep46030 (PMC5385541; doi:10.1038/srep46030)
Supplement: Supplementary Information [file srep46030-s1.pdf]

Maxdenominator Reweighted Sparse Representation for Tumor Classification

Weibiao Li, Bo Liao*, Wen Zhu, Min Chen, Li Peng, Xiaohui Wei, Changlong Gu, Keqin Li

Supplementary information includes:

Supplemental Information includes codes of the MRSRC and the introduction of the datasets.

Codes:

main function:

```
clc;
clear all;
close all;
addpath data;
fprintf(1,'Computing...\n');

FilterMethod = 0;
dataSet=1;
SelectFeaNum =30*30;

ResultAcc_MRSRC = {};

while dataSet <=9

switch dataSet
     case {1}
        load Colon.mat;
        dataSet=dataSet+1;
    case {2}
        load AMLALL.mat;
        dataSet=dataSet+1;
    case {3}
        load DLBCF.mat;
        dataSet=dataSet+1;
    case {4}
        load  Gliomas.mat;
        dataSet=dataSet+1;
    case {5}
        load SRBCT.mat;
        dataSet=dataSet+1;
    case {6}
        load ALL.mat;
        dataSet=dataSet+1;
    case {7}
        load MLLLeukemia.mat;
```

```
        dataSet=dataSet+1;
    case {8}
        load LeukemiaGloub.mat;
        dataSet=dataSet+1;

end
%========================================
TotalSet   = [TrainingSet; TestingSet];
TotalLabel = [TrainingLabel; TestingLabel];

%=======================================




SubClassNum  = CountingSubClassNum(TotalLabel);
minSubClassNum=min(SubClassNum);
MaxTimes=10;

SeAcc_MRSRC= [];


for iSubClassNum= 5:minSubClassNum - 1

    FiAcc_MRSRC= [];

    NumPerSubClass = iSubClassNum;
    for iTimes=1:MaxTimes
        TrainingSet=[];
        TrainingLabel=[];
        TestingSet=[];
        TestingLabel=[];


        [TrainingSet TrainingLabel TestingSet TestingLabel] =
HoldOutTrainingTest(TotalSet,TotalLabel,NumPerSubClass);

        if FilterMethod==2
                idx_top = relieff(TrainingSet, TrainingLabel, 5);
                IX = idx_top(:);
                TopGene = size(IX);
                TrainingSet = TrainingSet(:,IX(1:TopGene));
                TestingSet = TestingSet(:,IX(1:TopGene));
                TopGene = SelectFeaNum;
```

```
              TrainingSet = TrainingSet(:,1:TopGene);
              TestingSet = TestingSet(:,1:TopGene);
        end

        TrainingSet=mapstd(TrainingSet, 0, 1) ;
        TestingSet=mapstd(TestingSet, 0, 1) ;

        FiAcc_MRSRC = [FiAcc_MRSRC remeta_re(TrainingSet', TestingSet',
TrainingLabel, TestingLabel, iSubClassNum, .1)];

    end


    FiAcc_MRSRC= [FiAcc_MRSRC mean(FiAcc_MRSRC) std(FiAcc_MRSRC)];

    SeAcc_MRSRC= [SeAcc_MRSRC; FiAcc_MRSRC];

end

ResultAcc_MRSRC{dataSet-1} = {SeAcc_MRSRC};

end
save('result.mat','ResultAcc_MRSRC');
```

CountingSubClassNum function:

```
function [ SubClassNum ] = CountingSubClassNum(TotalLabel)
%UNTITLED2 Summary of this function goes here
%   Detailed explanation goes here

ClassNum  = max(TotalLabel);
SampleNum = size(TotalLabel,1);
SubClassNum = zeros(ClassNum,1);
for i = 1:SampleNum
    k=TotalLabel(i);
    SubClassNum(k,1)=SubClassNum(k,1)+1;
end

return
```

HoldOutTrainingTest function:

```
function [TrainingSet TrainingLabel TestingSet TestingLabel] =
HoldOutTrainingTest(TotalSet,TotalLabel,NumPerClass)
%UNTITLED Summary of this function goes here
```

```
%   Detailed explanation goes here
%ClassNum = max(TotalLabel);
ClassNum = length(unique(TotalLabel));
[SampleNum GeneNum] = size(TotalSet);
TrainingTestingDivision = zeros(SampleNum,1);

RandSamples = randperm(SampleNum)';
TotalSet = TotalSet(RandSamples,:);
TotalLabel = TotalLabel(RandSamples,:);
for i=1:ClassNum
   TempNumPerClass=0;
   for j=1:SampleNum
       if TotalLabel(j,1)==i
          TrainingTestingDivision(j,1)=1;
          TempNumPerClass=TempNumPerClass+1;
       end
       if TempNumPerClass==NumPerClass
          break;
       end
   end
end
TrainingSet  =TotalSet(find(TrainingTestingDivision==1),:);
TrainingLabel=TotalLabel(find(TrainingTestingDivision==1),:);
TestingSet   =TotalSet(find(TrainingTestingDivision==0),:);
TestingLabel =TotalLabel(find(TrainingTestingDivision==0),:);


[SampleP LabelIDX]=sort(TrainingLabel);
TrainingSet=TrainingSet(LabelIDX,:);
TrainingLabel = TrainingLabel(LabelIDX,:);

[SampleP LabelIDX] = sort(TestingLabel);
TestingSet  = TestingSet(LabelIDX,:);
TestingLabel = TestingLabel(LabelIDX,:);


return
```

remeta_re function:

```
function [ classrate, ids, decval ] = remeta_re( traindata, testdata,
trainlabels, testlabels, Numeigv, lamda )
%UNTITLED Summary of this function goes here
%   Detailed explanation goes here
classids = unique(trainlabels);
```

```
NumClass = length(classids);

traindata1=[];
trainlabels1=[];

for k = 1:NumClass
   % Numeigv = 0;
    [eigvector{1,k}, eigvalue] = svd(traindata(:,find(trainlabels==k)), 0);
   % Numeigv = size(eigvector{1,k}, 2);
    traindata1=[traindata1,eigvector{1,k}(:,1:Numeigv)];
    Tlabels(1:Numeigv,k)=k;
    trainlabels1=[trainlabels1; Tlabels(1:Numeigv,k)];
end

 traindata=traindata1;
 trainlabels=trainlabels1;
 decval = [];
 ids = [];
 for j = 1:size(testdata, 2)
     y = testdata(:, j);
     [id, s, err] = RE_MSRCRE(traindata, trainlabels, y, lamda);%和复权
     ids(j) = id;
     decval = [decval, err];
 end

classrate = sum(ids(:) == testlabels(:))/ length(testlabels);
```

RE_MSRCRE function:

```
function [id, s, minerr] = RE_MSRCRE(traindata, trainlabels, x, lambda)
% Use the extended SRC to classify data under occlusion

maxIter=10;
eeps=0.1;
s=pinv(traindata)*x;
for iter = 1:maxIter
     if iter > 1
          weights = 1./(abs(s)+eeps);
     else
           weights = 1*ones(size(traindata,2),1);
     end
     s=s.*weights;

     [s,status] = l1_ls(traindata, x,
```

```
lambda); %%%http://www.stanford.edu/~body/l1_ls
     %s=s./weights;
     x=traindata*s;
end

classids = unique(trainlabels);
NumClass = length(classids);

r = zeros(NumClass, 1);
for i = 1:NumClass
    classidx = find(trainlabels == classids(i));
    classdata = traindata(:, classidx);
    si = s(classidx);
    err = norm(classdata*si - x)/sum(si.^2);
    r(i) = err;
end

[minerr, id] = min(r);

id = classids(id);
```

introduction of the datasets:

Supplementary Dataset 1 is Colon dataset. It includes 4 tables :training set、training label、testing set、testing label.

Supplementary Dataset 2 is DLBCL dataset. It includes 4 tables :training set、training label、testing set、testing label.

Supplementary Dataset 3 is SRBCT dataset. It includes 4 tables :training set、training label、testing set、testing label.

Supplementary Dataset 4 is LeukemiaGloub dataset. It includes 4 tables :training set、training label、testing set、testing label.
